# Single-agent maintenance therapy for advanced non-small cell lung cancer (NSCLC): a systematic review and Bayesian network meta-analysis of 26 randomized controlled trials

**DOI:** 10.7717/peerj.2550

**Published:** 2016-10-20

**Authors:** Qinxue Wang, Haobin Huang, Xiaoning Zeng, Yuan Ma, Xin Zhao, Mao Huang

**Affiliations:** 1 Department of Respiratory & Critical Care Medicine, the First Affiliated Hospital of Nanjing Medical University, Nanjing, Jiangsu, China; 2 Department of Cardiovascular Surgery, the First Affiliated Hospital of Nanjing Medical University, Nanjing, Jiangsu, China

**Keywords:** Non-small cell lung cancer, Maintenance therapy, Bayesian network meta-analysis

## Abstract

**Background:**

The benefit of maintenance therapy has been confirmed in patients with non-progressing non-small cell lung cancer (NSCLC) after first-line therapy by many trials and meta-analyses. However, since few head-to-head trials between different regimens have been reported, clinicians still have little guidance on how to select the most efficacious single-agent regimen. Hence, we present a network meta-analysis to assess the comparative treatment efficacy of several single-agent maintenance therapy regimens for stage III/IV NSCLC.

**Methods:**

A comprehensive literature search of public databases and conference proceedings was performed. Randomized clinical trials (RCTs) meeting the eligible criteria were integrated into a Bayesian network meta-analysis. The primary outcome was overall survival (OS) and the secondary outcome was progression free survival (PFS).

**Results:**

A total of 26 trials covering 7,839 patients were identified, of which 24 trials were included in the OS analysis, while 23 trials were included in the PFS analysis. Switch-racotumomab-alum vaccine and switch-pemetrexed were identified as the most efficacious regimens based on OS (HR, 0.64; 95% CrI, 0.45–0.92) and PFS (HR, 0.54; 95% CrI, 0.26–1.04) separately. According to the rank order based on OS, switch-racotumomab-alum vaccine had the highest probability as the most effective regimen (52%), while switch-pemetrexed ranked first (34%) based on PFS.

**Conclusions:**

Several single-agent maintenance therapy regimens can prolong OS and PFS for stage III/IV NSCLC. Switch-racotumomab-alum vaccine maintenance therapy may be the most optimal regimen, but should be confirmed by additional evidence.

## Introduction

Lung cancer is one of the most common malignant tumors and the leading cause of cancer-related death worldwide. It is estimated that about 224,390 new cases of lung and bronchus cancer will be diagnosed and 158,080 deaths will occur in 2016 in the United States alone (Siegel, Miller & Jemal, 2016). Non-small cell lung cancer (NSCLC) accounts for approximately 85% of all lung cancer cases (Cufer, Ovcaricek & O’Brien, 2013).

For early stage NSCLC, radical surgery or radiotherapy may result in relatively better prognosis. Unfortunately, most patients (accounting for >70%) have advanced disease at diagnosis, thus are not amenable to curative treatment and are candidates for systemic therapy only, with a dismal 5-year survival rate of <5% (Rossi et al., 2014; Tan et al., 2015). The past decade has seen the evolution of individualized systemic treatment for advanced NSCLC. The development of the anti-cancer agents, especially the blossom of molecular targeted anti-tumor agents, has prolonged progression free survival (PFS) and overall survival (OS) of some selected patients with specific and sensitive gene mutations (Cufer, Ovcaricek & O’Brien, 2013). Nonetheless, as these mutations only occur in a small percentage of patients, 4–6 cycles of platinum-based double-agent chemotherapy is still the gold-standard regimen recommended by guidelines for most patients with non-resectable, locally-advanced, or metastatic NSCLC (Azzoli et al., 2011; Besse et al., 2014; Ettinger et al., 2015). Additionally, prolonged platinum-based doublet therapies show increased toxicity and no meaningful improvement in OS (Lima et al., 2009). In the past, patients who successfully responded to front-line therapy had to wait for disease progression before receiving second-line or other treatment, and unfortunately, nearly half of them could not proceed with second-line therapy, mostly due to their declining performance status (PS) (Berge & Doebele, 2013). In recent years, more attention has been focused on maintenance therapy, which refers to the extension of one or more agents to non-progressing patients after first-line induction chemotherapy (Owonikoko, Ramalingam & Belani, 2010).

Although relatively new for NSCLC, maintenance therapy has been used in the treatment of hematologic malignancies for years (Childhood ALL Collaborative Group, 1996; Salles et al., 2011). Continued use of at least one of the drugs given in induction therapy is defined as continuation maintenance, whereas switch maintenance refers to administration of a totally different agent from first-line chemotherapy (Ettinger et al., 2015). Switch-pemetrexed therapy has shown improvement of both OS and PFS compared to placebo as single-agent maintenance in Ciuleanu’s trial (Ciuleanu et al., 2009), as well as switch-erlotinib in Cappuzzo’s trial (Cappuzzo et al., 2010); and both have been approved by Food and Drug Administration for maintenance therapy of advanced NSCLC patients non-progressing after 4 cycles of platinum-based first-line chemotherapy in the United States (Cohen et al., 2010a; Cohen et al., 2010b). Former meta-analyses studies have confirmed that single-agent maintenance therapy can prolong OS and PFS in contrast to non-maintenance regimens (Behera et al., 2012; Yuan et al., 2012; Zhang et al., 2015). Factors that may predict beneficial effects from maintenance therapy include tumor histology, PS, and epidermal growth factor receptor (EGFR) mutation status (Zhou et al., 2015).

The recent inclusion of various agents such as sunitinib, pazopanib, and some vaccines in anti-tumor therapy, has propelled research into their use as maintenance therapy options for NSCLC (Ahn et al., 2013; Alfonso et al., 2014; Butts et al., 2005; Giaccone et al., 2015; O’Brien et al., 2015; Socinski et al., 2014). Some classic randomized clinical trials (RCTs) of maintenance therapy, such as the INFORM study (Zhao et al., 2015), have updated their final survival statistics as well. Nevertheless, the relative effects of any of these maintenance regimens compared with other regimens remain unclear due to lack of evidence from head-to-head RCTs. Network meta-analysis (NMA) can simultaneously synthesize evidence from both direct and indirect comparisons of diverse regimens into a single network, which enables us to estimate the relative efficacy of several agents when head-to-head RCTs are not available (Salanti et al., 2008). By adopting Bayesian approach in the analysis, we can rank the relative efficacy of these regimens by calculating the corresponding probability of OS (Ades et al., 2006). Hence, herein, we present a NMA to assess the efficacy of various single-agent maintenance therapy strategies for stage III/IV NSCLC. It is our belief that this analysis will provide some clinical evidence for clinicians to make decisions on maintenance therapy for NSCLC.

## Methods

### Search strategy

We conducted a comprehensive literature search of PubMed, EMBASE, and the Cochrane Central Register of Controlled Trials (CENTRAL) from inception to November 09, 2015. We administered a high sensitivity searched strategy with keywords set around “non-small cell lung cancer,” “maintenance therapy,” and “RCT.” No language restriction was administered. Details of the search strategy are presented in Supplemental Information 1. We also manually searched proceedings of the annual American Society of Clinical Oncology (ASCO) meetings and European Society of Medical Oncology (ESCO) congresses from 2000 to 2015 as a supplement. Citations of relevant reviews and trials were also screened. All results were input into Endnote X7 reference software (Thomson Reuters, Stamford, CT, US) for duplication exclusion and further reference management.

### Selection of trials

Studies meeting the following inclusion criteria were eligible: (i) RCTs, (ii) patients were pathologically or cytologically-diagnosed with non-resectable stage III or IV NSCLC, (iii) comparisons had to be between single-agent maintenance therapy and placebo, observation, or another single-agent maintenance regimen, and (iv) sufficient data on OS or/and PFS. Trials with randomization conducted before induction therapy and trials of complementary medicine were excluded. When multiple publications reported on one trial, we selected the most recent report for data extraction.

### Data extraction and risk of bias assessment

Data was independently extracted by two reviewers (Q Wang and H Huang) using standardized data compilation forms. Name of the first author, publication year, number of patients and population characteristics, induction and maintenance therapy regimens, survival statistics, adverse effects (AEs) were major aspects included. The hazard ratios (HRs) and 95% confidence intervals (CIs) were either obtained from the original articles or estimated from Kaplan–Meier curves using Tierney’s spreadsheet (Tierney et al., 2007). For each included trial, the following domains of bias were judged and ranked into “low risk,” “high risk,” or “unclear risk”: generation of random sequence, allocation concealment, blinding, incomplete outcome data, selective reporting of outcome, and other biases. Two investigators (Q Wang and H Huang) independently performed the assessment. All divergences during data extraction and assessment of risk of bias were solved by discussion with a third investigator (M Huang).

### Statistical analysis

The NMA combined evidence from head-to-head comparisons into a network to obtain estimates of the relative efficacy of each treatment. Analyses were conducted using R 3.0.1 (R Development Core Team, 2013) with an interface to WinBUGS 1.4.3 (Medical Research Council Biostatistics Unit, Cambridge, UK). We built a network within the Bayesian framework and the posterior distribution of the treatment effect was estimated using Markov Chain Monte Carlo methods.

For the three-arm trial *Perol 2012*, log HRs (contrast statistics) were converted to log HRs (arm-specific statistics) according to the method introduced by Woods, Hawkins & Scott (2010).

All analyses were performed with 2 chains, and each had a sample of 200,000 simulations after discarding the results of a burn-in period of 40,000 simulations. We estimated the relative treatment effects based on the posterior distributions and ranked the probability for each treatment in descending order as the most efficacious regimen, the second, the third, and so on, according to OS and PFS separately. Since OS is the most concerned outcome in clinical trials of antitumor therapy, we set OS as the primary outcome in our analysis and draw conclusions based on OS mainly. The deviance information criterion (DIC) provided a measure of model fit that a lower value suggested a simpler model. Convergence of the model was assessed with the Brooks–Gelman–Rubin diagnostic methods in WinBUGS.

Both fixed and random effects models were administered in the primary analysis; posterior mean of the residual deviance (resdev), effective number of parameters (pD), and DIC results of the two models were compared in sensitivity analysis. We assessed the inconsistency between direct and indirect evidence using the method suggested by Veroniki et al. (2013). We also assessed the probability of publication bias with contour-enhanced funnel plots (Peters et al., 2008).

### Quality assessment of evidence

We assessed the quality of evidence in two steps. First, we used the Grading of Recommendations Assessment, Development, and Evaluation (GRADE) system to assess quality of direct evidence (Guyatt et al., 2011). GRADE focuses on a body of evidence rather than individual studies. RCTs were initially identified as high quality of evidence and identification of problems on limitations in trial design, inconsistency, indirectness, imprecision and publication bias decreased the evidence quality rating. Quality of evidence was rated as high, moderate, low or very low. Then, we used the iGRADE approach, which is a modification of the GRADE approach for mixed treatment comparisons proposed by Dumville et al. (2012), to evaluate the quality of NMA evidence.

**Figure 1:**
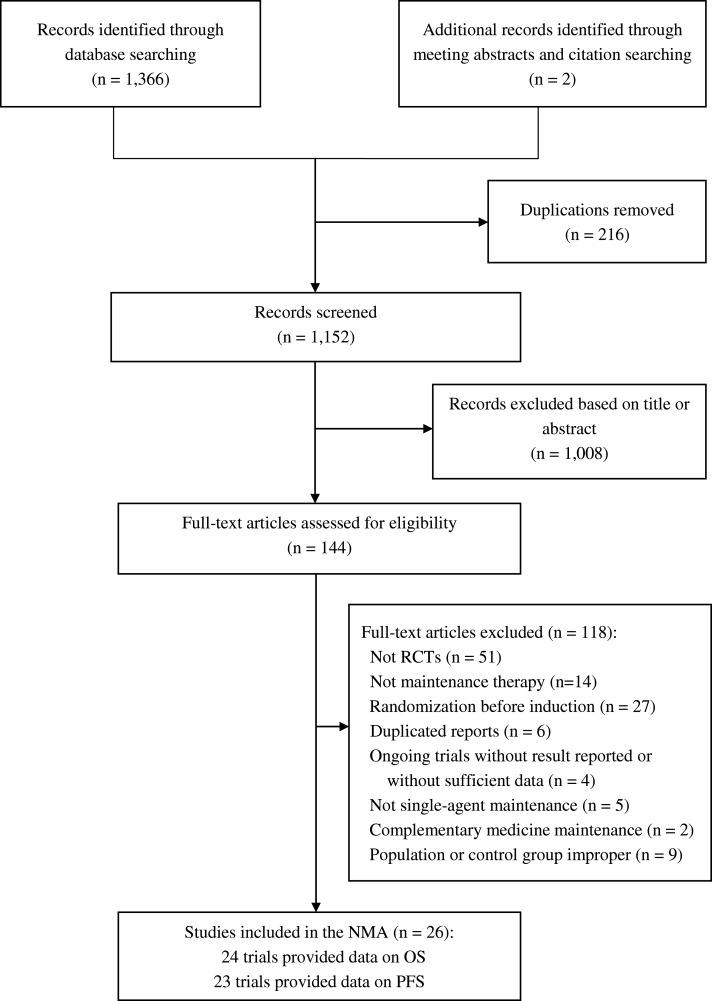
Flow diagram of trial selection. RCT, randomized controlled trial; NMA, network meta-analysis; OS, overall survival; PFS, progression free survival.

**Table 1:** General characteristics of the eligible RCTs.

Name/year (study name)	Number of maintenance		Population	Induction therapy	Maintenance therapy	Median age (years)	Males (%)	Squamous cell carcinoma (%)	HR (95% CrI)	
	T	C							OS	PFS
Belani 2003	65	65	CT-naïve, stage IIIB/IV NSCLC, ECOG PS 0-2	Paclitaxel + carboplatin	Con-pac 70 mg/m^2^ weekly for 3 of 4 weeks; Observation	65.5	81.3	NR	1.21^a^ (0.72–2.03)	/
Butts 2005	88	83	CT-naïve, stage IIIB/IV NSCLC, ECOG PS 0-2	Platinum-based CT alone or CT + radiotherapy	Swi-BLP 1,000 µg weekly for 8 weeks + BSC; BSC	59	55.6	NR	0.75^b^ (0.53–1.04)	/
Westeel 2005	91	90	CT-naïve, stage IIIB/ IV NSCLC, WHO PS 0-2	MIC + cisplatin (+ radiotherapy for IIIB)	Swi-vin 25 mg/m^2^ weekly for 6 months; Observation	62.5	92.8	59.7	1.08^b^ (0.79–1.47)	0.77^b^ (0.56–1.07)
Brodowicz 2006	138	68	CT-naïve, stage IIIB/ IV NSCLC, KPS ≥ 70	Gemcitabine + cisplatin	Con-gem 1,250 mg/m^2^ on days 1 & 8 of a 21-day cycle until PD or unacceptable toxicity; BSC	57.3	73.3	40.8	0.84^a^ (0.52–1.37)	0.69^a^ (0.56–0.86)
Hanna 2008	73	74	CT-naïve, unresectable stage IIIA/IIIB NSCLC, ECOG PS 0-1	Etoposide + cisplatin	Swi-doc 75 mg/m^2^ every 3 weeks for 3 cycles; Observation	62	70.1	NR	1.06^a^ (0.75–1.50)	1.01^a^ (0.77–1.33)
Johnson 2008	94	92	CT-naïve, stage III/IV NSCLC, ECOG PS 0-2	Platinum-based CT	Swi-CAI 250 mg/d until PD or unacceptable toxicity; Placebo	65.8	57.5	18.3	1.03^a^ (0.77–1.37)	1.02^a^ (0.82–1.27)
Kelly 2008 (SWOG S0023)	118	125	CT-naïve, unresectable stage IIIA/IIIB NSCLC, ECOG PS 0-1	Etoposide + cisplatin + radiotherapy	Swi-gef 500 mg/d for 5 years or until PD or unacceptable toxicity; Placebo	61.5	63.0	24.7	0.63^c^ (0.44–0.91)	0.80^c^ (0.58–1.10)
Ciuleanu 2009 (JMEN)	441	222	CT-naïve, stage IIIB/IV NSCLC, ECOG PS 0-1	Platinum-based CT (not include pemetrexed)	Swi-pem 500 mg/m^2^ on day 1 of a 21-day cycle; Placebo	60.5	72.9	27.5	0.79^c^ (0.65–0.95)	0.50^c^ (0.42–0.61)
Fidias 2009	153	156	CT-naïve, stage IIIB/IV NSCLC, ECOG PS 0-2	Gemcitabine + carboplatin	Swi-doc 75 mg/m^2^ on day 1 of a 21-day cycle until PD (maximum of 6 cycles); BSC + delayed docetaxel 75 mg/m^2^ on day 1 of a 21-day cycle (maximum of 6 cycles) once PD;	65.5	62.1	17.5	/	0.71^c^ (0.55–0.92)
Belani 2010	128	127	CT-naïve, stage IIIB/IV NSCLC, ECOG PS 0-1	Gemcitabine + carboplatin	Con-gem 1,000 mg/m^2^ on days 1 & 8 of a 21-day cycle until PD + BSC; BSC	67.0	NR	NR	0.97^c^ (0.72–1.30)	0.97^c^ (0.92–1.04)
Cappuzzo 2010 (SATURN; BO18192)	438	451	CT-naïve recurrent or stage IIIB/IV NSCLC, ECOG PS 0-1	Platinum-based CT	Swi-erl 150 mg/d until PD or unacceptable toxicity; Placebo	60	74.1	40.6	0.81^c^ (0.70–0.95)	0.71^c^ (0.62–0.82)
Hu 2010	33	30	CT-naïve, unresectable stage IIIA/IIIB NSCLC, PS 0-1	Platinum-based CT + radiotherapy	Swi-vin 21 mg/m^2^ on days 1 & 8 of a 28-day cycle for 6 cycles; Observation	56.7	58.7	46.0	0.89^a^ (0.55–1.43)	/
Gaafar 2011 (EORTC 08021/ILCP 01/03)	87	86	CT-naïve, stage IIIB/IV NSCLC, WHO PS 0-2	Platinum-based CT	Swi-gef 250 mg/d; Placebo	61.0	77.0	20.0	0.83^c^ (0.60–1.15)	0.61^c^ (0.45–0.83)
Carter 2012	61	58	CT-naïve, unresectable stage IIIA/IIIB NSCLC, ECOG PS 0-1	Paclitaxel + carboplatin + radiotherapy	Con-pac 70 mg/m^2^ weekly for 3 of 4 weeks for 6 months; Observation	63.5	33.6	23.5	1.22^a^ (0.75–1.99)	1.51^a^ (1.04–2.19)
Mubarak 2012	61	59	CT-naïve, stage IIIB/IV non-squamous NSCLC, ECOG PS 0-1	Pemetrexed + cisplatin	Con-pem 500 mg/m^2^ of a 21-day cycle until PD or unacceptable toxicity+ BSC; BSC	60.0	67.3	0	0.95^c^ (0.46–1.97)	0.65^c^ (0.35–1.20)
Perol 2012 (IFCT-GFPC 0502)	154	155	CT-naïve, stage IIIB/IV NSCLC, ECOG PS 0-1	Gemcitabine + cisplatin	Con-gem 1,250 mg/m^2^ on days 1 & 8 of a 21-day cycle; Swi-erl 150 mg/d; Observation	58.3	73.0	19.6	0.89^c^ (0.62–1.28)	0.56^c^ (0.44–0.72)
	155								0.87^c^ (0.68–1.13)	0.69^c^ (0.54–0.88)
Zhang 2012 (INFORM; C-TONG 0804)	148	148	CT-naïve, stage IIIB/IV NSCLC, WHO PS 0-2	Platinum-based CT	Swi-gef 250 mg/d; Placebo	55.0	40.9	19.3	0.88^b^ (0.68–1.14)	0.42^b^ (0.33–0.55)
Ahn 2013 (NCT00777179)	75	42	CT-naïve, stage IIIB or IV NSCLC, WHO PS 0-1	Gemcitabine + cisplatin	Swi-van 300 mg/d + BSC; Placebo + BSC	61.0	64.1	17.1	1.43^a^ (0.77–2.65)	0.75^a^ (0.53–1.05)
Karayama 2013	26	/	CT-naïve, stage IIIB/IV non-squamous NSCLC, ECOG PS 0-1	Pemetrexed + carboplatin	Con-pem 500 mg/m^2^ on day 1 of a 21-day cycle; Swi-doc 60 mg/m^2^ on day 1 of a 21-day cycle	65.0	74.1	0	1.27^c^ (0.50–3.33)	1.79^c^ (0.93–3.57)
	25									
Paz-Ares 2013 (PARAMOUNT)	359	180	CT-naïve, stage IIIB/IV non-squamous NSCLC, ECOG PS 0-1	Pemetrexed + cisplatin	Con-pem 500 mg/m^2^on day 1 of a 21-day cycle + BSC; Placebo + BSC	61.0	58.1	0	0.78^c^ (0.64–0.96)	0.62^c^ (0.49–0.79)
Alfonso 2014	87	89	CT-naïve, stage IIIB/IV non-squamous NSCLC, ECOG PS 0-2	Platinum-based CT (+ radiotherapy)	Swi-rac 1 mg, 5 immunizations every 2 weeks and reimmunizations every 4 weeks for 1 year; Placebo	NR	67.0	37.5	0.63^c^ (0.46–0.87)	0.73^c^ (0.53–0.99)
Butts 2014 (START)	829	410	CT-naïve, unresectable stage IIIA/IIIB NSCLC, ECOG PS 0-1	Platinum-based CT + radiotherapy	Swi-BLP weekly for 8 weeks and then every 6 weeks until PD; Placebo	61.2	68.3	46.2	0.88^b^ (0.75–1.03)	0.87^b^ (0.75–1.00)
Socinski 2014 (CALGB 30607)	106	104	CT-naïve, stage IIIB/IV non-squamous NSCLC, ECOG PS 0-1	Platinum-based CT	Swi-sun 37.5 mg/d; Placebo	66.0	55.7	33.2	1.08^a^ (0.78–1.52)	0.59^a^ (0.32–1.21)
Cai 2015	7	7	CT-naïve, stage IIIB/IV EGFR gene-mutated NSCLC, PS 0-2	Paclitaxel + cisplatin	Swi-gef 250 mg/d; Observation	61.0	53.3	0	/	0.60^a^ (0.03–11.33)
Giaccone 2015	270	262	CT-naïve, unresectable stage IIIA/IIIB/IV NSCLC, ECOG PS 0-2	Platinum-based CT (+ radiotherapy)	Swi-bel monthly for 18 cycles followed by 2 quarterly cycles; Placebo	61.0	57.7	27.4	0.94^c^ (0.73–1.20)	0.99^c^ (0.82–1.20)
O’Brien 2015 (EORTC 08092)	50	52	CT-naïve, stage IIIB/IV NSCLC, WHO PS 0-2	Platinum-based CT	Swi-paz 800 mg/d; Placeb	64.4	45.1	19.6	0.72^c^ (0.40–1.28)	0.67^c^ (0.43–1.03)

**Notes.**

a Unadjusted HRs estimated from Kaplan–Meier curves using Tierney’s spreadsheet.

b Adjusted HRs obtained from the original articles.

c Unadjusted HRs obtained from the original articles.

Abbreviations BSC best support care NR not reported ECOG Eastern Cooperative Oncology Group CT chemotherapy L-BLP25 tecemotide MIC mitomycin C KPS Karnofsky performance status PD progressive disease EGFR epidermal growth factor receptor OS overallsurvival PFS progression free survival HR hazard ratio CrI credible interval swi-pem switch-pemetrexed con-pem continue-pemetrexed swi-gef switch-gefitinib con-gem continue-gemcitabine swi-erl switch-erlotinib swi-doc switch-docetaxel con-pac continue-paclitaxel swi-BLP switch-L-BLP25 swi-bel switch-belagenpumatucel-L swi-paz switch-pazopanib swi-sun switch-sunitinib swi-van switch-vandetanib swi-CAI switch-carboxyaminoimidazole swi-vin switch-vinorelbine swi-rac switch-racotumomab-alum

## Results

### Characteristics of eligible studies

**Figure 2:**
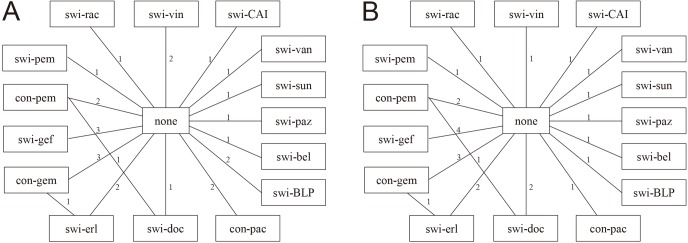
Network of evidence. (A) and (B) present network diagrams for OS and PFS separately. Numbers above the lines represent the amount of studies. Swi-pem, switch-pemetrexed; con-pem, continue-pemetrexed; swi-gef, switch-gefitinib; con-gem, continue-gemcitabine; swi-erl, switch-erlotinib; swi-doc, switch-docetaxel; con-pac, continue-paclitaxel; swi-BLP, switch-L-BLP25; swi-bel, switch-belagenpumatucel-L ; swi-paz, switch-pazopanib; swi-sun, switch-sunitinib; swi-van, switch-vandetanib; swi-CAI, switch-carboxyaminoimidazole; swi-vin, switch-vinorelbine; swi-rac, switch-racotumomab-alum.

Through online databases and meeting abstracts searches, a total of 1,368 records were identified. After rounds of assessment, 26 trials covering 7,839 patients met all the inclusion criteria, and comprised of 24 complete manuscripts (Ahn et al., 2013; Alfonso et al., 2014; Belani et al., 2003; Brodowicz et al., 2006; Butts et al., 2005; Butts et al., 2014; Cai et al., 2015; Cappuzzo et al., 2010; Carter et al., 2012; Ciuleanu et al., 2009; Fidias et al., 2009; Gaafar et al., 2011; Giaccone et al., 2015; Hanna et al., 2008; Hu et al., 2010; Johnson et al., 2008; Karayama et al., 2013; Kelly et al., 2008; Mubarak et al., 2012; O’Brien et al., 2015; Pérol et al., 2012; Paz-Ares et al., 2013; Westeel et al., 2005; Zhang et al., 2012) and 2 meeting abstracts (Belani et al., 2010; Socinski et al., 2014). Selection procedure is summarized in Fig. 1. Summary of characteristics of the 26 eligible studies and HR data of each individual study is shown in Table 1. With the exception of *Perol 2012* which was a three-arm trial (continue-gemcitabine or switch-erlotinib vs. observation) and *Karayama 2013* which compared two maintenance regimens directly (continue-pemetrexed vs. switch-docetaxel); the remaining 24 trials all compared single-agent maintenance therapy vs. no-maintenance control. The network of evidence constructed by the included RCTs is shown in Fig. 2. Risks of bias of the enrolled studies are depicted in Table S1.

### OS and PFS analyses

**Figure 3:**
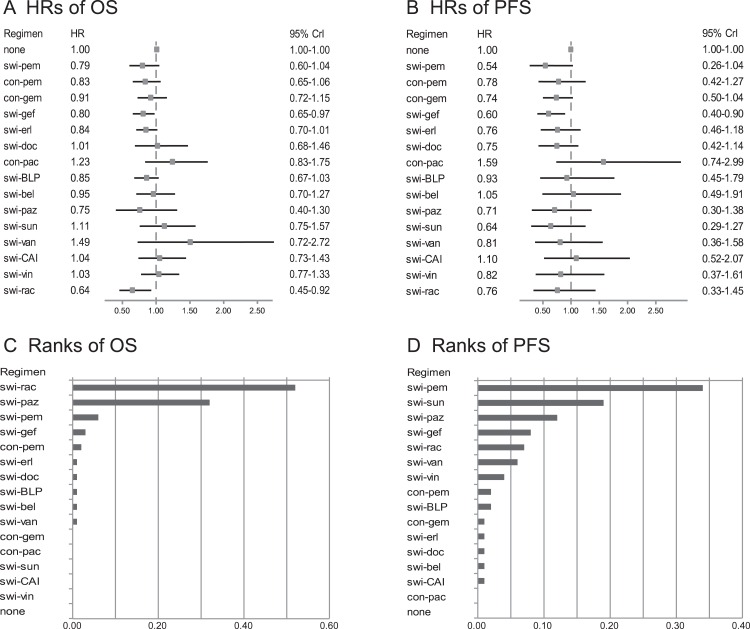
OS and PFS analyses in total population. (A) and (B) show comparisons of HRs based on OS and PFS respectively in an unselected population. Switch-racotumomab-alum vaccine showed most excellent efficacy compared to no-maintenance with a HR = 0.64 (95% CI [0.45–0.92]) in OS analysis, as well as switch-pemetrexed (HR, 0.54; 95% CI [0.26–1.04]) in PFS analysis. (C) and (D) show the probability of every regimen to be the best one based on OS and PFS respectively in an unselected population. According to the rank order based on OS, switch-racotumomab-alum vaccine came first (52%). Based on PFS, switch-pemetrexed ranked first (34%). Swi-pem, switch-pemetrexed; con-pem, continue-pemetrexed; swi-gef, switch-gefitinib; con-gem, continue-gemcitabine; swi-erl, switch-erlotinib; swi-doc, switch-docetaxel; con-pac, continue-paclitaxel; swi-BLP, switch-L-BLP25; swi-bel, switch-belagenpumatucel-L; swi-paz, switch-pazopanib; swi-sun, switch-sunitinib; swi-van, switch-vandetanib; swi-CAI, switch-carboxyaminoimidazole; swi-vin, switch-vinorelbine; swi-rac, switch-racotumomab-alum; OS, overall survival; PFS, progression free survival; HR, hazard ratio; CrI, credible interval.

In total, 24 trials were included in the OS analysis (Ahn et al., 2013; Alfonso et al., 2014; Belani et al., 2003; Belani et al., 2010; Brodowicz et al., 2006; Butts et al., 2005; Butts et al., 2014; Cappuzzo et al., 2010; Carter et al., 2012; Ciuleanu et al., 2009; Fidias et al., 2009; Gaafar et al., 2011; Giaccone et al., 2015; Hanna et al., 2008; Hu et al., 2010; Johnson et al., 2008; Karayama et al., 2013; Kelly et al., 2008; Mubarak et al., 2012; O’Brien et al., 2015; Pérol et al., 2012; Paz-Ares et al., 2013; Westeel et al., 2005; Zhang et al., 2012). No-maintenance control was set as the reference in all analyses. Based on assessment of model fit, results calculated by random effects models are presented in this section. The HRs for different maintenance regimens compared to no-maintenance are shown in Fig. 3A. Several maintenance therapy regimens yielded longer OS than no-maintenance, although differences were not statistically significant in some regimens. Switch-docetaxel, continue-paclitaxel, switch-sunitinib, switch-vandetanib, switch-carboxyaminoimidazole (CAI), and switch-vinorelbine did not improve OS. Switch-maintenance therapy with racotumomab-alum vaccine showed excellent efficacy compared to no-maintenance with a HR = 0.64 [95% credible intervals (CrI), 0.45–0.92] Pooled relative treatment effect estimates of all comparisons are presented in Table S2.

In PFS analysis, we included 23 trials (Ahn et al., 2013; Alfonso et al., 2014; Belani et al., 2010; Brodowicz et al., 2006; Butts et al., 2014; Cai et al., 2015; Cappuzzo et al., 2010; Carter et al., 2012; Ciuleanu et al., 2009; Fidias et al., 2009; Gaafar et al., 2011; Giaccone et al., 2015; Hanna et al., 2008; Johnson et al., 2008; Karayama et al., 2013; Kelly et al., 2008; Mubarak et al., 2012; O’Brien et al., 2015; Pérol et al., 2012; Paz-Ares et al., 2013; Socinski et al., 2014; Westeel et al., 2005; Zhang et al., 2012). The HRs for different maintenance regimens compared to no-maintenance regimens are shown in Fig. 3B. Continue-paclitaxel, switch-belagenpumatucel-L, or switch-CAI did not yield longer PFS than no-maintenance. Switch-pemetrexed and switch-gefitinib showed excellent efficacy compared to no-maintenance with HRs = 0.54 (95% CI [0.26–1.04]) and 0.60 (95% CI [0.40–0.90]). Pooled relative treatment effect estimates of all comparisons are presented in Table S2.

Ranking which indicated the probability of the best regimen in descending order, among all treatments is shown in Figs. 3C, 3D and Table 2. According to the rank order based on OS, switch-racotumomab-alum vaccine had the greatest probability as the best regimen (52%), with switch-pazopanib ranked second (32%), and switch-pemetrexed ranked third (6%). Based on PFS, switch-pemetrexed ranked first (34%), followed by switch-sunitinib (19%), with switch-pazopanib ranked third (12%).

**Table 2:** Rank orders.

Regimen	R 1	R 2	R 3	R 4	R 5	R 6	R 7	R 8	R 9	R 10	R 11	R 12	R 13	R 14	R 15	R 16
**OS**																
none	0.00	0.00	0.00	0.00	0.00	0.00	0.00	0.02	0.09	0.18	0.28	0.26	0.12	0.04	0.01	0.00
swi-pem	0.06	0.15	0.18	0.15	0.12	0.11	0.06	0.05	0.04	0.03	0.02	0.01	0.02	0.00	0.00	0.00
con-pem	0.02	0.01	0.14	0.13	0.11	0.12	0.12	0.08	0.08	0.04	0.03	0.01	0.02	0.01	0.00	0.00
con-gem	0.00	0.02	0.04	0.04	0.06	0.10	0.11	0.16	0.13	0.12	0.09	0.05	0.04	0.03	0.01	0.00
swi-gef	0.03	0.12	0.17	0.18	0.16	0.11	0.09	0.06	0.04	0.02	0.01	0.01	0.01	0.00	0.00	0.00
swi-erl	0.01	0.05	0.10	0.12	0.15	0.15	0.13	0.13	0.06	0.05	0.03	0.02	0.01	0.00	0.00	0.00
swi-doc	0.01	0.02	0.04	0.04	0.05	0.05	0.06	0.06	0.07	0.08	0.09	0.09	0.12	0.10	0.10	0.04
con-pac	0.00	0.00	0.00	0.01	0.01	0.02	0.02	0.02	0.02	0.04	0.03	0.08	0.10	0.15	0.31	0.21
swi-BLP	0.01	0.04	0.08	0.13	0.14	0.14	0.15	0.11	0.08	0.07	0.03	0.02	0.01	0.01	0.00	0.00
swi-bel	0.01	0.02	0.03	0.05	0.06	0.07	0.09	0.10	0.10	0.08	0.09	0.09	0.08	0.09	0.04	0.01
swi-paz	0.32	0.20	0.08	0.05	0.05	0.03	0.04	0.04	0.04	0.02	0.03	0.02	0.03	0.03	0.02	0.02
swi-sun	0.00	0.01	0.01	0.02	0.02	0.01	0.02	0.04	0.05	0.08	0.07	0.07	0.13	0.19	0.19	0.08
swi-van	0.01	0.01	0.01	0.01	0.02	0.01	0.01	0.01	0.03	0.02	0.02	0.02	0.05	0.08	0.13	0.57
swi-CAI	0.00	0.02	0.02	0.03	0.03	0.03	0.05	0.06	0.06	0.08	0.10	0.12	0.14	0.14	0.10	0.04
swi-vin	0.00	0.01	0.01	0.02	0.01	0.03	0.06	0.06	0.10	0.10	0.10	0.13	0.16	0.13	0.08	0.03
swi-rac	0.52	0.25	0.08	0.04	0.03	0.03	0.01	0.01	0.01	0.00	0.01	0.01	0.00	0.00	0.00	0.00
**PFS**																
none	0.00	0.00	0.00	0.00	0.00	0.00	0.00	0.00	0.02	0.05	0.12	0.23	0.28	0.22	0.07	0.00
swi-pem	0.34	0.19	0.12	0.09	0.06	0.05	0.04	0.03	0.03	0.02	0.01	0.01	0.01	0.01	0.01	0.00
con-pem	0.02	0.04	0.05	0.07	0.08	0.08	0.10	0.09	0.10	0.10	0.10	0.07	0.03	0.05	0.02	0.01
con-gem	0.01	0.02	0.05	0.07	0.10	0.11	0.14	0.12	0.14	0.11	0.07	0.03	0.02	0.01	0.01	0.00
swi-gef	0.08	0.16	0.18	0.16	0.13	0.09	0.06	0.05	0.04	0.02	0.01	0.01	0.01	0.00	0.00	0.00
swi-erl	0.01	0.04	0.05	0.08	0.07	0.11	0.11	0.14	0.10	0.08	0.08	0.06	0.03	0.02	0.02	0.00
swi-doc	0.01	0.03	0.07	0.07	0.09	0.11	0.12	0.12	0.09	0.09	0.07	0.05	0.04	0.02	0.02	0.00
con-pac	0.00	0.00	0.00	0.00	0.00	0.00	0.01	0.01	0.01	0.03	0.03	0.03	0.04	0.08	0.16	0.61
swi-BLP	0.02	0.01	0.03	0.03	0.05	0.05	0.07	0.07	0.07	0.09	0.08	0.10	0.09	0.09	0.10	0.04
swi-bel	0.01	0.01	0.02	0.02	0.03	0.03	0.04	0.04	0.05	0.08	0.07	0.09	0.11	0.15	0.16	0.11
swi-paz	0.12	0.14	0.08	0.10	0.08	0.06	0.05	0.06	0.05	0.05	0.06	0.05	0.04	0.04	0.04	0.01
swi-sun	0.19	0.13	0.12	0.08	0.08	0.07	0.06	0.04	0.06	0.04	0.03	0.02	0.02	0.02	0.02	0.01
swi-van	0.06	0.07	0.06	0.06	0.08	0.07	0.06	0.06	0.07	0.07	0.09	0.06	0.05	0.06	0.06	0.02
swi-CAI	0.01	0.01	0.02	0.02	0.03	0.03	0.04	0.03	0.04	0.06	0.07	0.08	0.10	0.13	0.21	0.13
swi-vin	0.04	0.07	0.07	0.06	0.05	0.08	0.05	0.07	0.07	0.06	0.07	0.07	0.07	0.07	0.07	0.03
swi-rac	0.07	0.08	0.09	0.09	0.08	0.07	0.06	0.06	0.06	0.07	0.07	0.05	0.06	0.04	0.04	0.02

**Notes.**

Abbreviations R rank OS overallsurvival PFS progression free survival HR hazard ratio CrI credible interval swi-pem switch-pemetrexed con-pem continue-pemetrexed swi-gef switch-gefitinib con-gem continue-gemcitabine swi-erl switch-erlotinib swi-doc switch-docetaxel con-pac continue-paclitaxel swi-BLP switch-L-BLP25 swi-bel switch-belagenpumatucel-L swi-paz switch-pazopanib swi-sun switch-sunitinib swi-van switch-vandetanib swi-CAI switch-carboxyaminoimidazole swi-vin switch-vinorelbine swi-rac switch-racotumomab-alum

### Adverse events (AEs)

Maintenance chemotherapy (including pemetrexed, gemcitabine, docetaxel, paclitaxel, and vinorelbine) was commonly associated with hematologic events such as neutropenia, thrombocytopenia, and anemia. Maintenance tyrosine kinase inhibitor (TKI) (including EGFR-TKI and other TKIs) commonly caused more skin and gastrointestinal AEs, such as rash, nausea, and vomiting. Maintenance vaccine (including belagenpumatucel-L, racotumomab-alum, and L-BLP25) was commonly associated with injection site reaction and flu-like symptoms. The main AE of CAI was nausea.

### Sensitivity analysis

The primary outcome OS was calculated using both fixed and random effects models. Resdev, pD and DIC were very similar for both models (−23.37, 15.8 and −7.5 in fixed effects model; −23.33, 17.3 and −6.0 in random effects model), which indicated the robustness of results.

### Inconsistencies

**Figure 4:**
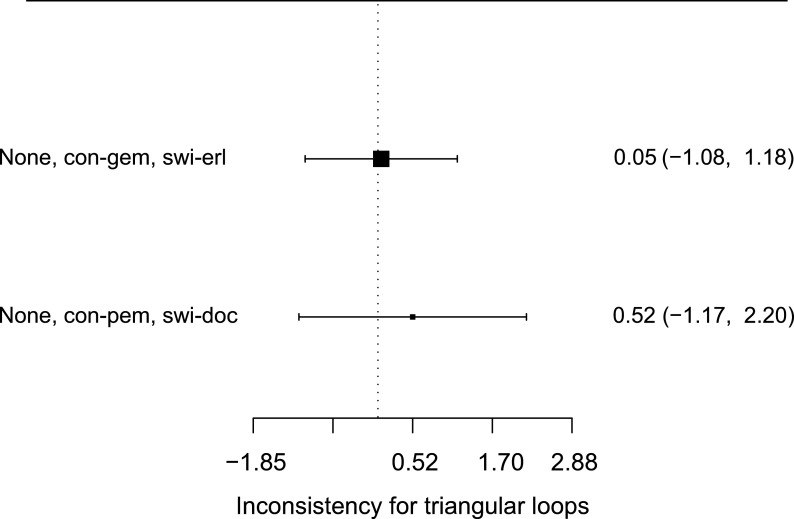
Inconsistencies evaluation (based on OS). Only two closed loops were formed (none vs. con-gem vs. swi-erl; none vs. con-pem vs. swi-doc) in this NMA. The size of the black square represented the amount of included studies. Both loops had their credible intervals covered blank value, which meant there was no evidence of inconsistencies between direct and indirect data.

The data did not suggest any inconsistency between direct and indirect evidence in the network (Fig. 4). In fact, direct evidence of the relative efficacy of different maintenance therapy regimens was rather few in the network. In the analysis of OS, only two closed loops were formed (none vs. continue-gemcitabine vs. switch-erlotinib; none vs. continue-pemetrexed vs. switch-docetaxel).

### Publication bias

**Figure 5:**
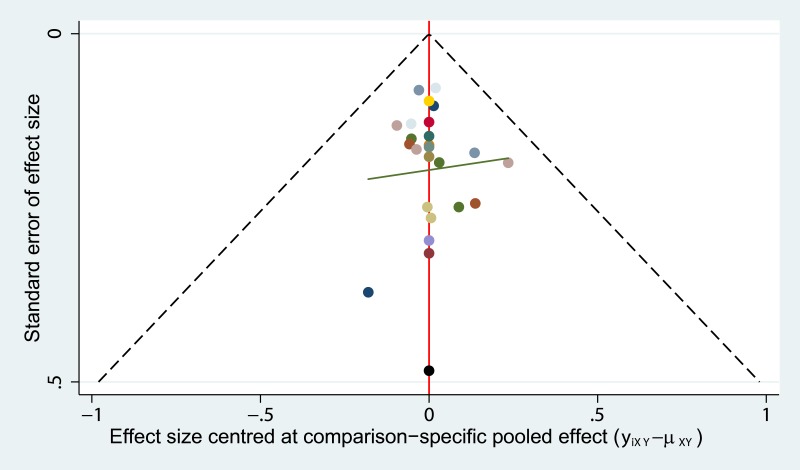
Publication bias (based on OS). The funnel plot did not suggest any publication bias in the network.

Symmetry of the ‘comparison-adjusted’ funnel plot suggested that efficacy of the regimens were no more exaggerated than their respective comparison-specific weighted average effect in small studies. The regimens also sorted from oldest to newest, and the resulting ‘comparison-adjusted’ funnel plot did not suggest any publication bias in the network (Fig. 5).

### Quality assessment

GRADE and iGRADE analyses are presented in Table S3. Since direct data comparing different maintenance therapy regimens was available for only two couples of regimens, measurement of inconsistencies between direct and indirect data was limited. In general, the most common reasons for lowering the quality of evidence were limitations in trial design and imprecision in some studies. Data suggested that evidence on switch-docetaxel, continue-paclitaxel and switch-vinorelbine were rated as limited quality, while evidence on switch-pemetrexed, switch-belagenpumatucel-L and switch-racotumomab-alum was rated as higher quality.

## Discussion

Although recent evidence provided by both RCTs and meta-analyses have shown that maintenance therapy might improve the outcomes of patients without progressive disease (PD) after induction treatment, there is still little guidance on the choice of the most suitable regimens for patients with different characteristics in clinical practice. This NMA study compared the survival benefits among all available single-agent maintenance regimens based on OS and PFS in unselected population with the aim of providing beneficial information for making clinical decisions for NSCLC maintenance therapy.

Comparing to chemotherapy as well as EGFR-TKIs, cancer vaccines are a relative new treatment strategy but show promise in NSCLC therapy. NeuGcGM3 gangliosides are normally expressed on the plasma membranes of mammalian cells except human cells, due to a 92-bp deletion in the human gene that encodes an enzyme which catalyzes the conversion of N-acetyl to NeuGc sialic acid (Alfonso et al., 2014). However, NeuGcGM3 gangliosides are over-expressed on several tumor cells membranes, such as melanoma, breast cancer, and NSCLC. In addition, NeuGcGM3 gangliosides also play important roles in tumor biology, including promoting tumor metastasis and reinforcing tumor immune escape. Furthermore, NSCLC patients with higher expression of NeuGc gangliosides have lower OS and PFS. All the above characteristics make NeuGc gangliosides attractive targets for tumor immunotherapy (Hernández & Vázquez, 2015). Racotumomab-alum vaccine is an anti-idiotype vaccine targeting the NeuGcGM3 tumor-associated gangliosides, which can bind and directly kill NSCLC cells expressing the antigen. According to our NMA in unselected population, switch-racotumomab-alum vaccine might be the most efficacious maintenance regimen in prolonging OS. Switch-racotumomab-alum can decrease the hazard for death to 0.64. However, since there was only one study (176 patients) on racotumomab-alum vaccine, additional RCT studies with larger patient populations are required to confirm this finding. At present, racotumomab-alum vaccine is marketed in Cuba and Peru as maintenance therapy for NSCLC patients. Meanwhile, clinical researches are underway in the United Kingdom and China.

Switch-pemetrexed and switch- pazopanib maintenance therapy also revealed favorable effect in prolonging OS. Pemetrexed has shown different effects according to pathological category of NSCLC, and is extremely efficacious for non-squamous NSCLC. In Ciuleanu’s study, switch-pemetrexed maintenance therapy decreased the HRs for OS and PFS to 0.70 (95% CI [0.56–0.88]) and 0.44 (95% CI [0.36–0.55]) in non-squamous population, which was significantly better than in the squamous population (OS HR 1.07, 95% CI [0.77–1.50]; and PFS HR 0.69, 0.49–0.98) (Ciuleanu et al., 2009). Therefore, switch-pemetrexed may be an efficacious regimen for non-squamous NSCLC. However, these two maintenance regimens have each been investigated in single eligible studies, therefore additional studies are required to confirm these observations.

Our NMA still has several limitations. Firstly, some regimens had few trials eligible for analysis, thus their small sample sizes may influence the reliability of outcomes. Secondly, since different agents and regimens have their particular target population, treating all unselected NSCLC patients as a whole may lead to the underestimation of some efficacious regimens. Thirdly, single bevacizumab maintenance therapy as a potential effective regimen has been investigated in AVAPERL (Barlesi et al., 2011) and ATLAS (Johnson et al., 2013) trials. However, since both of those two studies did not incorporate no-maintenance therapy as control, we could not integrate them into the network, thus the efficacy of bevacizumab maintenance was not compared to the other regimens.

Survival outcomes of patients receiving maintenance therapy are influenced by post-study therapy. However, most of the included studies did not provide detailed information about the effect of post-study therapy on survival. Some studies have reported that though maintenance therapy could improve PFS, there were no significant differences in OS (Ahn et al., 2013; Pérol et al., 2012). We supposed the nonconformity between PFS and OS results may partly be due to the choice of different post-study therapy. Thus, in our NMA, we also set PFS as an outcome to analysis. Future studies could choose maintenance regimen with PFS benefit continued with different post-maintenance therapies to determine which combination has the best OS outcome. Apart from efficacy and safety, the quality of life of patients and the cost-effectiveness of maintenance therapy should be taken into consideration when choosing maintenance therapy. Future studies should incorporate all these aspects in their study design and analysis.

In conclusion, our NMA demonstrates that several single-agent maintenance therapy regimens may prolong OS and PFS for stage III/IV NSCLC. Racotumomab-alum vaccine has shown potential survival benefit in unselected NSCLC population but should be confirmed with additional clinical evidence.

##  Supplemental Information

10.7717/peerj.2550/supp-1 Table S1 Risk of bias

10.7717/peerj.2550/supp-2 Table S2 Pooled relative treatment effects

10.7717/peerj.2550/supp-3 Table S3 Quality assessment

10.7717/peerj.2550/supp-4 Supplemental Information 1 Search strategy

10.7717/peerj.2550/supp-5 Supplemental Information 2 PRISMA checklist

10.7717/peerj.2550/supp-6 Data S1 Raw data extracted from all eligible studies (including basic information of the studies, baseline of the population, survival outcomes, and adverse events) applied for data analyses and preparation for Table 1

## Additional Information and Declarations

### Data Availability

The following information was supplied regarding data availability: The raw data has been supplied as a Supplementary File.
