# Supplementary material for: Single-agent maintenance therapy for advanced non-small cell lung cancer (NSCLC): a systematic review and Bayesian network meta-analysis of 26 randomized controlled trials
Source: PeerJ. 2016 Oct 20;4:e2550. doi: 10.7717/peerj.2550 (PMC5075715; doi:10.7717/peerj.2550)
Supplement: Supplemental Information 1 [file peerj-04-2550-s004.pdf]

Search strategies for PubMed, EMBASE, CENTRAL database

PubMed search details: 515 results

((((((("randomized controlled trial"[Publication Type] OR "randomized controlled trials as topic"[MeSH Terms] OR "randomized controlled trial"[All Fields] OR "randomised controlled trial"[All Fields]) OR ("controlled clinical trial"[Publication Type] OR "controlled clinical trials as topic"[MeSH Terms] OR "controlled clinical trial"[All Fields])) OR ("random allocation"[MeSH Terms] OR ("random"[All Fields] AND "allocation"[All Fields]) OR "random allocation"[All Fields] OR "randomized"[All Fields])) OR randomly[All Fields]) OR ("clinical trials as topic"[MeSH Terms] OR ("clinical"[All Fields] AND "trials"[All Fields] AND "topic"[All Fields]) OR "clinical trials as topic"[All Fields] OR "trial"[All Fields])) OR groups[All Fields]) NOT (("animals"[MeSH Terms:noexp] OR animals[All Fields]) NOT ("humans"[MeSH Terms] OR "humans"[All Fields]))) AND ((consolidation[All Fields] AND ("therapy"[Subheading] OR "therapy"[All Fields] OR "therapeutics"[MeSH Terms] OR "therapeutics"[All Fields])) OR (("maintenance"[MeSH Terms] OR "maintenance"[All Fields]) AND ("therapy"[Subheading] OR "therapy"[All Fields] OR "therapeutics"[MeSH Terms] OR "therapeutics"[All Fields])))) AND (((("lung"[MeSH Terms] OR "lung"[All Fields]) AND (non-small[All Fields] AND ("cells"[MeSH Terms] OR "cells"[All Fields] OR "cell"[All Fields]))) OR ("carcinoma, non-small-cell lung"[MeSH Terms] OR ("carcinoma"[All Fields] AND "non-small-cell"[All Fields] AND "lung"[All Fields]) OR "non-small-cell lung carcinoma"[All Fields] OR "nsclc"[All Fields])) OR "Carcinoma, Non-Small-Cell Lung"[Mesh])

EMBASE: 632 results

No.	Query Results	Results	Date
#11.	'non small cell lung cancer'/exp OR nsclc OR (non AND small AND cell AND lung AND cancer) OR ('non small' AND cell AND lung AND cancer) AND	632	9 Nov 2015

	(consolidation AND therapy OR (maintenance AND therapy) OR 'maintenance therapy'/exp) AND ('controlled clinical trial'/de OR 'randomized controlled trial'/de)		
#10.	'non small cell lung cancer'/exp OR nsclc OR (non AND small AND cell AND lung AND cancer) OR ('non small' AND cell AND lung AND cancer) AND (consolidation AND therapy OR (maintenance AND therapy) OR 'maintenance therapy'/exp)	10,388	9 Nov 2015
#9.	consolidation AND therapy OR (maintenance AND therapy) OR 'maintenance therapy'/exp	1,102,684	9 Nov 2015
#8.	'non small cell lung cancer'/exp OR nsclc OR (non AND small AND cell AND lung AND cancer) OR ('non small' AND cell AND lung AND cancer)	101,421	9 Nov 2015
#7.	'maintenance therapy'/exp	1,024,838	9 Nov 2015
#6.	maintenance AND therapy	106,820	9 Nov 2015
#5.	consolidation AND therapy	14,232	9 Nov 2015
#4.	'non small' AND cell AND lung AND cancer	77,601	9 Nov 2015
#3.	non AND small AND cell AND lung AND cancer	81,525	9 Nov 2015
#2.	nsclc	41,554	9 Nov 2015
#1.	'non small cell lung cancer'/exp	87,341	9 Nov 2015

CENTRAL: 219 results

#1 MeSH descriptor: [Carcinoma, Non-Small-Cell Lung] explode all trees 2559

#2 nsclc 3456

#3 non-small cell lung cancer 5420

#4 consolidation therapy 1314

#5 MeSH descriptor: [Maintenance Chemotherapy] explode all trees 127

#6 Maintenance Chemotherapy 2118

#7 #1 or #2 or #3 5822

#8 #4 or #5 or #6 3150

#9 #7 and #8 in Trials 219
